# Quantification and comparison of 4D Flow MRI derived wall shear stress and MRE derived wall shear stiffness of abdominal aorta

**DOI:** 10.1186/1532-429X-18-S1-P360

**Published:** 2016-01-27

**Authors:** Venkata Sita Priyanka Illapani, Julio Garcia, Ria Mazumder, Richard D White, Michael Markl, Arunark Kolipaka

**Affiliations:** 1 grid.261331.40000000122857943Department of Radiology, The Ohio State University, Columbus, OH USA; 2 grid.465264.7Department of Radiology, Northwestern University, Chicago, IL USA; 3 grid.261331.40000000122857943Department of Internal Medicine Division of Cardiology, The Ohio State University, Columbus, OH USA; 4 grid.261331.40000000122857943Department of Electrical and Computer Engineering, The Ohio State University, Columbus, OH USA; 5 grid.261331.40000000122857943Department of Biomedical Engineering, The Ohio State University, Columbus, OH USA; 6 grid.465264.7Department of Biomedical Engineering, Northwestern University, Columbus, OH USA

## Background

Aortic wall shear stiffness (AWS) and wall shear stress (WSS) are important indicators of pathological changes in the abdominal aorta. Previous studies have shown that AWS increases with diseases such as hypertension and atherosclerosis while WSS decreases^1,2^ with these diseases. Therefore, early detection of AWS and WSS could significantly impact timely treatment of these pathological conditions. Non-invasive estimation of AWS and WSS became feasible after the recent advent of phase contrast MRI based magnetic resonance elastography (MRE)^3^ and 4D flow MRI^4^ respectively. We hypothesize that investigating the relationship between AWS and WSS may provide additional information to assist in diagnosis of aortic diseases. Therefore, in this study, we use both MRE and 4D flow MRI to estimate and establish the correlation between AWS and WSS in normal human subjects.

## Methods

MRI was performed on 20 volunteers in a 3T Siemens scanner (Tim-Trio, Siemens Healthcare, Germany). Imaging parameters included: ***MRE****:* TE/TR = 9.52/14.28 ms; slice thickness = 6 mm; number of slices = 3; acquisition matrix = 128 × 64; α = 25^o^; Field of view (FOV) = 40 cm^2^; number of segments = 6 to 8 and a motion encoding gradient (MEG) = 120 Hz; ***4D PC MRI***: TE/TR = 2.1/5.1 ms; velocity encoding = 150cm/s; flip angle = 7^o^, acquisition matrix = 192 × 120 × 26; temporal resolution = 40.8 ms, spatial resolution = 1.7 × 2 × 2.2 mm^3^. Images were processed in MRElab (Mayo Clinic, Rochester, MN) to estimate end-systolic (ES) AWS while Ensight (CEI, Apex, NC) and custom built tool programmed in Matlab^5^ was used to measure WSS. Pulse wave velocity (PWV) was also estimated in Matlab (Mathworks, Natick, MA) to investigate the correlation with ES AWS.

## Results

**Figure 1:**
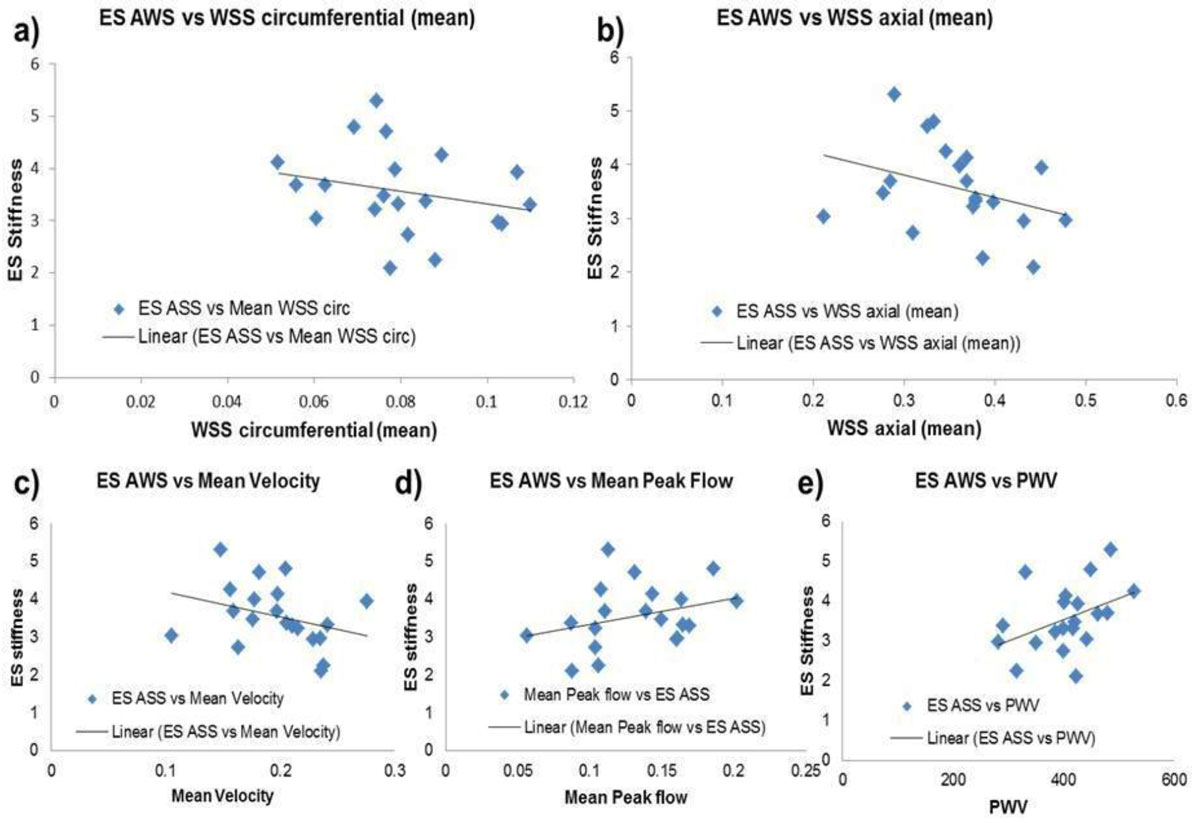
**Results - comparison between wall shear stress and stiffness**.

We observed a negative trend with no significant correlation between WSS (axial/circ) and ES AWS (Figure 1a,b). 4D flow derived WSS depend on fluid profile (i.e. laminar flow, peak flow and mean velocity) while MRE derived AWS is dependent on frequency of excitation, hence significant correlation fails to exist due to different principles being involved. Furthermore, very weak correlations were observed between ES AWS and PWV (R2 = 0.17), ES AWS and mean velocity (R2 = 0.1) and ES AWS and mean peak flow (R^2^ = 0.1) as shown in Figure 1c, d and e respectively. PWV and mean peak flow show a positive correlation with ES AWS while mean velocity shows a negative correlation.

## Conclusions

From our results we can conclude that there is no correlation between ES AWS and WSS, however more studies are warranted.

